# Genu Valgum, Fractures, and Renal Stones in a 10-year-old Girl

**DOI:** 10.1210/jcemcr/luac022

**Published:** 2022-12-02

**Authors:** Stephanie Christensen, Lindsey A Loomba

**Affiliations:** Department of Pediatrics, Section of Endocrinology, University of California Davis Medical Center, Sacramento, CA 95817, USA; Department of Pediatrics, Section of Endocrinology, University of California Davis Medical Center, Sacramento, CA 95817, USA

**Keywords:** rickets, hypophosphatemia, hypercalciuria, phosphorus, vitamin D

## Abstract

Rickets is a disorder of impaired bone mineralization that can arise from nutritional deficiencies and inherited conditions. We describe a 10-year-old girl presenting with genu valgum and a history of renal stones due to hereditary hypophosphatemic rickets with hypercalciuria (HHRH), a rare inherited form of rickets characterized by high 1,25 vitamin D levels, hypophosphatemia with inappropriate renal phosphate wasting, and hypercalciuria. After the diagnosis was confirmed, she began treatment with phosphorus supplementation and stopped taking vitamin D, leading to improved bone mineral density and reduction in renal symptoms. Patients with HHRH can be distinguished from those with other forms of hypophosphatemic rickets by their high 1,25 vitamin D levels in conjunction with low to normal parathyroid hormone and fibroblast growth factor 23 (FGF23) levels. Genetic testing for *SLC34A3* variants provides a definitive diagnosis.

Rickets is characterized by defects in growth plate mineralization, and can arise due to nutritional deficiencies, inherited conditions, or a combination thereof [1]. In the United States, diagnoses of rickets are on the rise. When left untreated, patients develop symptoms such as femur and tibial bowing, slowed growth, and bone pain. Depending on the underlying etiology, patients may also be at risk for a variety of other symptoms such as dental disease, hearing impairment, alopecia, and renal stones; thus, an accurate diagnosis is important for determining how to appropriately monitor and treat these patients [1-4].

Here, we describe a 10-year-old girl with a rare subtype of inherited rickets known as hereditary hypophosphatemic rickets with hypercalciuria (HHRH). This condition is inherited in an autosomal recessive manner through variants in the *SLC34A3* gene, which encodes for a sodium-phosphate cotransporter in the proximal renal tubule known as NaPi2c. NaPi2c stimulates reabsorption of phosphate and is counteracted by parathyroid hormone (PTH), which internalizes the NaPi2c receptor to cause phosphate wasting. In HHRH, the altered transporter function leads to renal phosphate loss even in the setting of hypophosphatemia, thus predisposing the patient to low bone mineral density and fractures [4, 5]. HHRH is a fibroblast growth factor 23 (FGF23)-independent process and FGF23 levels are normal or low, in contrast with many other inherited forms of rickets in which FGF23 participates in vitamin D and phosphate metabolism and regulation [1].

As a compensatory measure for phosphate wasting, the enzyme 1-alpha-hydroxylase is activated, leading to increased production of 1,25 vitamin D from 25 vitamin D and consequent elevation of 1,25 vitamin D levels. While helpful for attempting to raise serum phosphorus levels, the high 1,25 vitamin D has the additional effect of promoting calcium reabsorption from the intestine and kidney. Since calcium levels are generally normal in patients with HHRH, the excess calcium is consequently excreted through the urine, causing hypercalciuria and renal stones.

Given the wide range of symptom severity and onset among patients with HHRH, the diagnosis is difficult, increasing the likelihood of delay of appropriate treatment. In describing the case below, we outline a characteristic presentation of the condition and contrast the patient's findings with other forms of inherited rickets.

## Case Presentation

**Figure 1.**
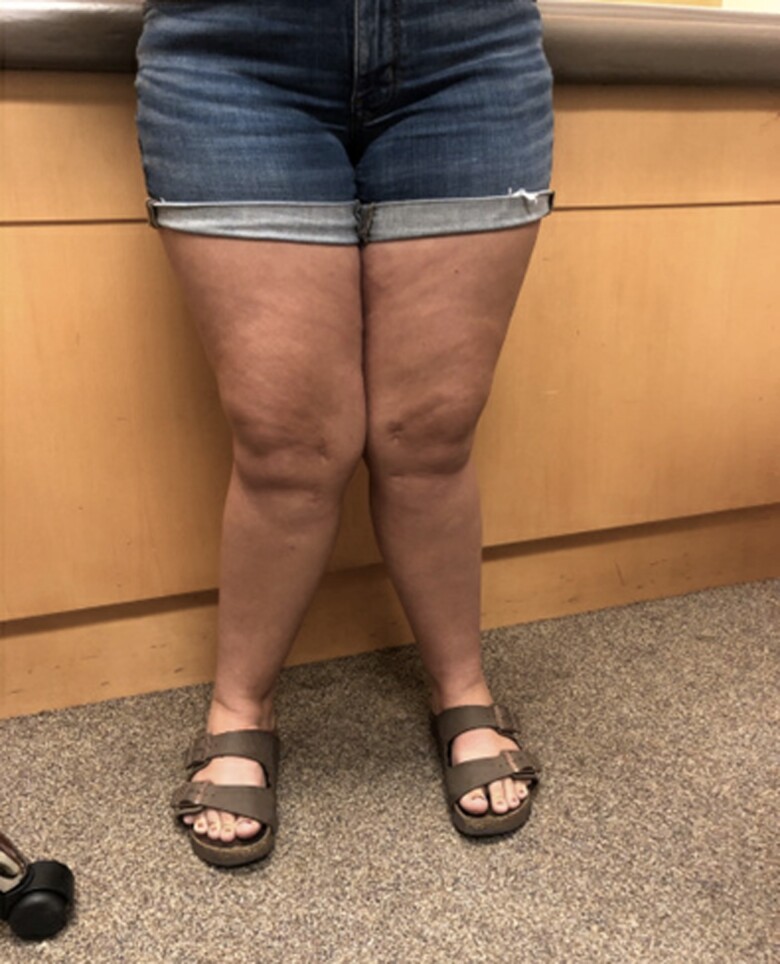
Genu valgum in adolescence.

A 10-year-old girl presented to our pediatric endocrinology office with a history of severe genu valgum and findings consistent with metabolic bone disease (Fig. 1). She had been diagnosed with possible vitamin D deficient rickets several weeks earlier by an orthopedic surgeon and referred to endocrinology for further evaluation. At the time of her first endocrinology visit, she was taking 400 IU daily of vitamin D. According to the patient's mother, the bowing of her legs became apparent at 5 years of age and did not interfere with normal motor development, so earlier intervention was not pursued. Her genu valgum was treated surgically by hemi-epiphysiodesis using 8-plate tethers 1 month after the initial endocrinology evaluation.

She had a prior history of hospitalization at the age of 3 years for a kidney stone, which she was able to pass spontaneously. The composition of the stone was unknown, and she had no further kidney stones or urinary symptoms since that time. She broke her right distal radius after a fall from playground equipment (“monkey bars”) when she was 8 years old. There was no family history of skeletal dysplasia, recurring fractures, bone disease, or renal stones. She denied symptoms of bone pain or muscle weakness.

## Diagnostic Assessment

Apart from significant genu valgum, her physical exam was normal, with no evidence of pectus abnormalities or joint swelling. Her growth curves were tracking appropriately; her weight was at the 45th percentile and height was at the 23rd percentile for age. A dual-energy X-ray absorptiometry scan showed a total hip Z-score of −2.0 and lumbar spine Z-score of 0.5, consistent with low bone mineral density.

Laboratory studies were obtained and showed hypophosphatemia (2.7 mg/dL [0.87 mmol/L]; reference range, 4.0-5.2 mg/dL [1.3-1.7 mmol/L]) and low parathyroid hormone (8 pg/mL [0.85 pmol/L]; reference range, 10-65 pg/mL [1.1-6.9 pmol/L]), along with high alkaline phosphatase (1213 U/L [21 µkat/L]; reference range, 129-417 U/L [2-7 µkat/L]) and high 1,25 vitamin D (>220 pg/mL [572 pmol/L]; reference range, 24-86 pg/mL [62-224 pmol/L]). Calcium (10.0 mg/dL [2.5 mmol/L]; reference range, 9.3-10.6 mg/dL [2.3-2.7 mmol/L]) and 25-hydroxy vitamin D were both normal (28.4 ng/mL [70.9 nmol/L]; reference range, 20-50 ng/mL [50-125 nmol/L]). Given her history of renal stones, a spot urine calcium to creatinine ratio was also obtained, which was elevated at 0.31 mg/mg (0.86 mmol/mmol); reference range < 0.25 mg/mg (< 0.7 mmol/mmol).

## Treatment

At the time of her initial endocrinology evaluation, the leading diagnosis was X-linked hypophosphatemic rickets. Accordingly, the patient's vitamin D supplementation was transitioned from 400 IU vitamin D daily to calcitriol 2 mcg daily and she started a 1000 mg elemental calcium supplement. Given the elevation in 1,25 vitamin D, vitamin D resistant rickets was also high on the differential, so molecular testing of the *VDR* (vitamin D receptor) gene was also obtained. No abnormalities were detected.

Follow-up testing for urine calcium and phosphorus excretion showed a low TMP/GFR (2.2 mg/dL; normal range, 2.9-6.5 mg/dL) and mild hypercalciuria (0.28 mg/mg [0.78 mmol/mmol]; reference range, < 0.25 mg/mg [< 0.7 mmol/mmol]). The patient was asked to stop her calcitriol and calcium supplements and began phosphorus supplementation with sodium-potassium-phosphate tablets 500 mg 3 times daily. Medication compliance was initially difficult and 6 months after beginning phosphorus supplementation, she rolled her ankle while walking in a swimming pool and sustained a possible Salter-Harris Type 1 fracture of the right distal fibula.

Additional genetic testing confirmed this patient's diagnosis of HHRH by revealing a compound heterozygous variant in the *SLC34A3* gene: c.575C>T (p.Ser192Leu), which she inherited from her mother, and c.925+20_926-48del (intronic), which she inherited from her father.

## Outcome and Follow-Up

Follow-up laboratory workup 4 months after beginning phosphorus showed persistent hypophosphatemia (2.5 mg/dL [0.81 mmol/L]; reference range, 4.0-5.2 mg/dL [1.3-1.7 mmol/L]) and improvements in her alkaline phosphatase (935 U/L [16 µkat/L]; reference range, 129-417 U/L [2-7 µkat/L]) and hypercalciuria (urine calcium to creatinine ratio 0.08 mg/mg [0.22 mmol/mmol]; reference range, < 0.25 mg/mg [< 0.7 mmol/mmol]). Her phosphorus supplementation was adjusted based on serum levels to 750 mg twice daily and she grew appropriately over the next several years, though her final adult height (148 cm, 1st percentile) was significantly less than predicted based on mid-parental height (166 cm, approximately 65th percentile). Bilateral medullary nephrocalcinosis was identified on a renal ultrasound; however, no further renal stones developed. Her bone mineral density also improved (total hip Z-score of −1.6 and lumbar spine Z-score of 0.6). She did not have further fractures.

## Discussion

The patient's genu valgum and poor bone mineralization immediately raised suspicion for rickets, of which there are several varieties (Table 1). The most common form globally is nutritional rickets, brought about by a deficiency of vitamin D and/or calcium [3]. Differentiating the various subtypes is possible by a combination of basic lab work and genetic testing to confirm gene variations.

**Table 1.** Differential diagnosis of hypophosphatemic rickets

	Gene	PTH	1,25 Vit. D	Urine Phosphorus	Urine Calcium
**Nutritional Vitamin D Deficiency**	—	High	Variable, with very low 25 Vit. D	High	Low
**XLH**	PHEX	Normal	Low	High	Low
**ADHR**	FGF23	Normal	Low/normal	High	Low
**ARHR**	DMP1/ENPP1	Normal	Low/normal	High	Low
**HHRH**	SLC34A3	Normal	High	High	High
**VDDR1A**	CYP27B1	High	Low	High	Low
**HVDR**	VDR	High	High	High	Low
**Dent Disease**	CLCN5	High	High	Normal/High	High

Abbreviations: ADHR, autosomal dominant hypophosphatemic rickets; ARHR, autosomal recessive hypophosphatemic rickets; HHRH, hereditary hypophosphatemic rickets with hypercalciuria; HVDR, hereditary vitamin D resistant rickets (formerly known as VDDR type 2); VDDR1A, vitamin D-dependent rickets type 1A; XLH, X-linked hypophosphatemic rickets

In the United States, inherited forms are the most prevalent form of rickets, with X-linked hypophosphatemic rickets (XLH) being the most common [2]. XLH arises secondary to genetic mutations in *PHEX*, which upregulates FGF23 expression. As FGF23 levels rise in patients with XLH, renal phosphate losses ensue and there is decreased conversion of 25 vitamin D into its active metabolite 1,25 vitamin D (calcitriol), leading to hypophosphatemia and low 1,25 vitamin D [2]. Other forms of FGF23-mediated rickets include autosomal dominant hypophosphatemic rickets (ADHR) and autosomal recessive hypophosphatemic rickets (ARHR) [1, 3].

Hereditary vitamin D resistant rickets (HVDR) is a rare condition which results in high levels of 1,25 vitamin D due to end-organ resistance, generally from mutations at the level of the *VDR* gene. PTH levels will be high, serum calcium is generally normal, and individuals tend to have alopecia or sparse body hair in conjunction with bony findings [3].

HHRH shares HVDR's constellation of low serum phosphorus and high 1,25-vitamin D but differs in that patients with HHRH exhibit an inappropriately elevated urine calcium and normal PTH. HHRH, as its name implies, is an inherited illness and 47 gene variants of *SLC34A3* have been implicated, the most common being c.575C>T (p.S192L) [4]. The patient described above had inherited from her mother this c.575C>T variant, which has been shown to significantly diminish NaPi2c's transporting capabilities once inserted into the membrane [6]. The variant c.925+20_926-48del, which was inherited from the patient's father, has been shown to alter a nucleotide within the intron that affects splicing, while not changing the ultimate SLC34A3 protein sequence [7-9]. Of note, the c.925+20_926-48del variant has also been referred to in the literature as g.2259-2359del [7, 8] or as a 101-bp deletion in intron 9 [9].

As an autosomal recessive condition, 2 variants are required for the disease to occur, making it important to gather a detailed family history looking into bone health, rickets, and renal stones. Heterozygous individuals may also demonstrate symptoms, especially renal symptoms such as renal stones and nephrocalcinosis, although their symptoms tend to be milder than patients with homozygous or compound heterozygous variants [4-6].

The treatment for HHRH consists of phosphorus supplementation in the form of phosphorus tablets. Unfortunately, these tablets must be taken multiple times daily and have a bad taste, which often results in poor adherence, as seen in this case. Since HHRH causes elevated 1,25 vitamin D and urine calcium levels, it is important to avoid vitamin D supplementation, unlike in many other forms of rickets, as it can worsen renal symptoms.

## Learning Points

- The differential diagnosis for rickets includes nutritional deficiencies, as well as inherited forms that encompass FGF23-mediated disease, variants in the vitamin D receptor and disorders of renal phosphate wasting.
- Symptoms of HHRH include bone pain, fractures, and bone deformities, along with renal complaints such as renal stones and nephrocalcinosis.
- Workup for rickets should include serum phosphorus, calcium, 1,25 vitamin D, PTH, and FGF23, if available, as well as urine calcium and phosphorus levels.
- HHRH is characterized by low serum phosphorus, high 1,25 vitamin D and elevated urine calcium. PTH and FGF23 levels are often low to normal, setting it apart from other forms of rickets.
- HHRH is treated with phosphorus supplementation and is associated with elevated calcitriol levels; thus, it is important to avoid excess vitamin D in these patients.

## Data Availability

Data sharing is not applicable to this article as no datasets were generated or analyzed during the current study.

## Abbreviations

FGF23: fibroblast growth factor 23
HHRH: hereditary hypophosphatemic rickets with hypercalciuria
HVDR: hereditary vitamin D resistant rickets
PTH: parathyroid hormone
XLH: X-linked hypophosphatemic rickets
